# A fast and reproducible cell- and 96-well plate-based method for the evaluation of P2X7 receptor activation using YO-PRO-1 fluorescent dye

**DOI:** 10.14440/jbm.2017.136

**Published:** 2017-01-20

**Authors:** Patrice Rat, Elodie Olivier, Caroline Tanter, Anaïs Wakx, Mélody Dutot

**Affiliations:** ^1^UMR 8638 CNRS COMETE, Université Paris Descartes, Sorbonne Paris Cité, Faculté de Pharmacie, 4 avenue de l’Observatoire, 75006 Paris, France; ^2^Soliance-Givaudan, Route de Bazancourt, 51110 Pomacle, France; ^3^Recherche et Développement, Laboratoire d’Evaluation Physiologique, Yslab, 2 rue Félix Le Dantec, 29000 Quimper, France

**Keywords:** cytometry, degenerescence, fluorescence, microplate, P2X7 receptor

## Abstract

The YO-PRO-1 assay provides a quantitative estimation of P2X7 receptor activation. P2X7 receptor is associated to pathological conditions including infectious, inflammatory, neurological, musculoskeletal disorders, pain and cancer. Most primary cells and cell lines from diverse origin may be used thanks to the ubiquitous distribution of P2X7 receptor. To study the activation of P2X7 receptor by chemicals or biological agents, we established a microplate-based cytometry protocol to accurately and rapidly quantify the activation of P2X7 receptor that leads to the formation of large pores in cell membranes. The YO-PRO-1 assay is based on the ability of cells to incorporate and bind YO-PRO-1 dye to DNA after activation of P2X7 receptor through pore formation. Cells are seeded in 96-well plates and incubated with the compound being tested for the appropriate time. The microplate is then incubated for 10 min with YO-PRO-1 staining solution. After the 10 min staining time, fluorescence signal is read using a microplate reader in 1 min. This procedure is easier and requires less handling steps than flow cytometry. 96-well plate based YO-PRO-1 assay is a reproducible and fast method to study both P2X7 receptor activation by toxic agents at subnecrotic concentrations and P2X7 receptor inhibition by antagonists.

## BACKGROUND

High throughput screening methods are emerging approaches in toxicology, allowing a quick and efficient toxicity evaluation through a battery of assays that target cellular processes [1]. These *in vitro* assays are useful for rapidly evaluating a large number of compounds and providing insight on potential human health effects [2]. These compounds include chemicals, drugs, plant extracts, environmental substances, formulations…

**Figure 1.**
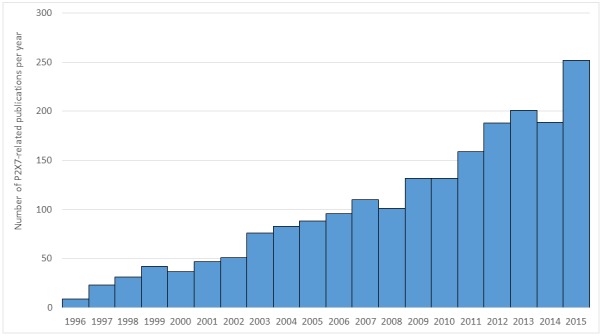
Number of P2X7-related publications per year (source: PubMed, keyword: P2X7).

P2X7 receptor, originally classified as P2Z receptor, has attracted more and more interest from the biomedical research community over the years, as shown in **Figure 1**, which has been generated by The “Results by year” PubMed timeline tool. Indeed, P2X7 receptor was discovered in 1996, which resulted in nine publications that year; almost twenty years later, more than 2240 publications are available on PubMed database, with 252 papers published in 2015.

P2X7 receptor is expressed by virtually all types of cells [3-5], including zebrafish, fish and frog cells [6-8]. This receptor belongs to P2X family ionotropic receptor binding ATP [9]. P2X7 receptors require higher concentrations of ATP for activation compared with other P2X receptors, and 2,3-O-(4-benzoylbenzoyl)-ATP (BzATP) is approximately 30-fold more potent than ATP in the human and rat [9, 10]. BzATP is extensively used in research as the agonist of choice for P2X7 receptor [11-13]. Brief stimulation of P2X7 receptor leads to an increase in intracellular calcium influx whereas repeated or prolonged (> 15 min) stimulation of P2X7 receptor induces the formation of a non-selective pore. This pore allows the entry of solutes up to 900 Da in size, which eventually leads to membrane blebbing, release of cytokines and activation of caspases ultimately leading to cell death [14-19]. P2X7 receptors activation is responsible for a large number of biological processes; consequently, P2X7 receptor has been associated to pathological conditions including infectious, inflammatory, autoimmune, neurological, musculoskeletal disorders, pain and, cancer [12, 20-27]. The role of P2X7 receptor in numerous pathologic conditions makes it a major therapeutic target, both in studies aiming at inhibiting P2X7 receptor and in studies aiming at activating P2X7 receptor (during the development of anticancer strategies).

### Development of the method

**Figure 2.**
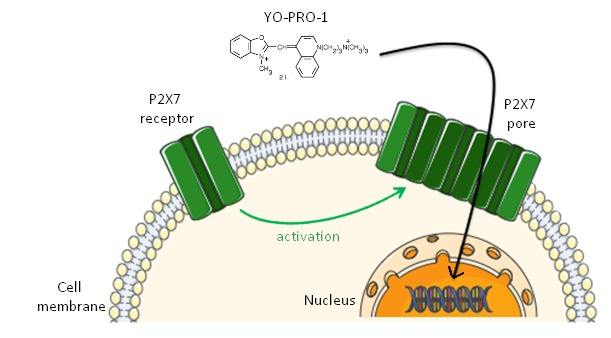
Schematic representation of YO-PRO-1 uptake after P2X7 receptor activation.

Through the formation of P2X7 pore, the cells become permeable for cationic dyes such as quinolinium,4-[(3-methyl-2(3H)-benzoxazolylidene)methyl]-1[3-(triethylammonio)propyl]-, diiodide (YO-PRO-1, 629 Daltons for the di-iodide salt; 375 Daltons for free base) [28, 29]. YO-PRO-1 is a cell membrane-impermeant cation whose fluorescence increases upon binding nucleic acids [30] and thus represents an excellent probe for detecting permeability changes in P2X7 receptor (**Fig. 2**).

**Figure 3.**
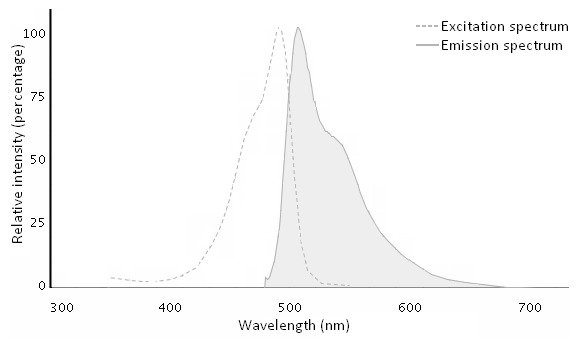
Fluorescence spectra of YO-PRO-1. Reprinted from Thermo Fisher Scientific website, retrieved September 15, 2016 from https://www.thermofisher.com/order/catalog/product/Y3603.

Originally, YO-PRO-1 signal was observed under fluorescence microscopy [9, 31] then quantified using microplate cytometry [32, 33] or flow cytometry [34]. The first studies looking at P2X7 activation through YO-PRO-1 uptake used fluorescence microplate readers with filter detection systems; we were among the first to use a monochromator-based system [35-43]. Excitation and emission wavelengths for YO-PRO-1 are very close, 491 nm and 509 nm, respectively (**Fig. 3**).

With monochromator-based readers, the wavelengths to be measured are generally available in 1-nm increments. This is very useful with fluorescent tags that have close excitation and emission wavelengths to avoid wavelengths overlap and then reduce background fluorescence and increase sensitivity and specificity of the tag.

Our 96-well plate-based method for the evaluation of P2X7 receptor activation using YO-PRO-1 fluorescent dye was developed for adherent living cells so the whole assay, from the incubation with the compounds being evaluated to the quantification of YO-PRO-1 signal, can be performed in the same 96-well plate. Minimizing sample handling procedures such as centrifugation and pipetting steps limits the risk to affect the levels of apoptotic body formation. This microtiter plate-based assay offers a simple, reproducible, fast, relatively inexpensive, accurate cell-based method for high throughput screening of numerous compounds (such as drugs, chemicals, pollutants, xenobiotics…) in their ability to activate P2X7 receptor and therefore cell death [36, 37, 44, 45]. It can also be used to study physiopathologic mechanisms implicated in degenerative diseases. Indeed, we previously used this technique to highlight the role of P2X7 receptor activation in age-related macular degeneration [2, 46]. Retinal cells (human retinal pigment epithelial ARPE-19 cell line and human retinal Müller glial MIO-M1 cell line) were incubated with amyloid β peptide or oxysterols, involved in degenerative diseases such as Alzheimer and age-related macular degeneration, then the YO-PRO-1 assay that we describe here was used to study P2X7 receptor activation. This assay allowed us to conclude that P2X7 receptor could be an important target to develop new treatments for age-related macular degeneration.

### Comparison with other techniques

Flow cytometry is not widely used as a screening tool due to the limitations in handling large numbers of samples and may not be as adapted as microplate cytometry for high throughput screening. Microplate cytometry is the quantitative analysis of cells in a microplate using a microplate reader. The use of microplates in cytometry provides opportunities to screen large numbers of samples.

### Experimental design

This protocol provides instructions for performing a cell-based procedure that quantifies the activation of P2X7 receptor directly in 96-well microplate. The procedure can be divided into three main parts: preparation of samples (Steps 1–15), preparation of stains and sample staining (Steps 16–17); and data acquisition by microplate cytometry and data analysis (Steps 18–19).

### Preparation of samples

Disruption of cell membrane integrity, a feature of necrosis, can lead to YO-PRO-1 entry in cells through necrotic pores [47]. In that case, increase in YO-PRO-1 uptake is not due to P2X7 receptor activation but to altered membrane integrity occurring during necrosis. To avoid misinterpretation of the results, YO-PRO-1 fluorescence signal has to be monitored on cells with preserved membrane integrity wherein YO-PRO-1 can only enter through activated P2X7 receptor [48]. Neutral red uptake assay for the estimation of cell viability through measurement of membrane integrity is highly recommended [49], but other cell viability assays can be used such as the Alamar Blue assay [50] or the MTT assay [51].

We developed the procedure for adherent cell lines (epithelial cells, macrophages, glial cells…) but it can be adapted to any primary cells, and also to suspension and non-adhesive cells (leukocytes, monocytes…) on condition that a centrifuge equipped with standard microplate holder is used. Cell lines obtained from various immortalization processes were tested: spontaneously immortalized cells, SV40 large T-antigen immortalized cells, and cancer cells. As YO-PRO-1 is light sensitive, clear bottom black 96-well microplates should be preferred. The cells are plated into 96-well microplates; it is worth noting that seeding densities are chosen to reach subconfluence in 24 h. The culture medium in which the cells are seeded is growth medium (generally supplemented with 10% FCS). The cells are incubated with the compounds suspected to activate P2X7 receptor directly in the microplate. The choice of the solution in which the compounds are diluted depends on the incubation time: for short incubation times (from 15 min to 1 h), the compounds can be diluted in phosphate buffer saline but for longer incubation times (superior to 1 hour), the compounds have to be diluted in maintenance medium (no more than 5% FCS, 2.5% is a good option for most cases) to avoid cell proliferation and reduction in sensitivity of chemicals with high protein affinity in 10% FCS.

Positive controls should be included in the assay to check the ability of agonists to activate P2X7 receptor in the chosen cell model. ATP (millimolar concentration) and BzATP (micromolar concentration) are the agonists of choice for YO-PRO-1 uptake studies.

If the assay is run on nonadherent cells, the microplate has to be centrifuged before any removal of cell supernatants. It should be noted that centrifugation will introduce a stage of sample handling that could influence the cell viability. Therefore, high centrifugation speed should be avoided (maximum 1800 rpm).

### Preparation of staining solution and sample staining

As divalent cations alter the affinity of ATP binding to the P2X7 receptor [29, 38, 52], it is of high importance to dilute YO-PRO-1 stock solution in phosphate buffer saline without calcium nor magnesium. After removal of the compound being evaluated, the cells are incubated with YO-PRO-1 dye directly in the microplate for only 10 min. It is noteworthy that neither fixation nor permeabilization are needed.

### Data acquisition by microplate cytometry

In the data acquisition software of the microplate reader, YO-PRO-1 excitation and emission wavelengths have to be specifically selected (491 nm, 509 nm) with the narrowest bandwidths. To ensure optimal fluorescence signal recording, it is crucial during data acquisition to let the microplate reader determine the optimal gain setting by selecting an automatic gain adjustment option. YO-PRO-1 fluorescence signal is monitored directly in the microplate without any washing step.

After YO-PRO-1 fluorescence signal quantitation, the cells can be observed by live-cell fluorescence microcopy technique and pictures can be taken to illustrate YO-PRO-1 uptake.

## MATERIALS

The indicated suppliers listed below can be substituted with appropriate alternatives.

### Reagents

• Cultured human corneal epithelial SV40 large T-antigen immortalized HCE cell line (Human Corneal Epithelial cells, Riken Cell Bank (RCB), cat. #1384)
• Cultured human retinal epithelial spontaneously immortalized ARPE-19 cell line (ATCC, cat. #CRL2302)
• Cultured human cutaneous epithelial spontaneously immortalized HaCaT cell line (Cell Lines Service, Batch #300493–2417)
• Cultured human placental trophoblast-derived choriocarcinoma JEG-3 cell line (ATCC, cat. #HTB-36)

We have also used this procedure on various primary cells and animal cell lines.

**NOTE**: The cell lines used in your research should be regularly checked to ensure that they are authentic and not infected with Mycoplasma. Mycoplasma contamination can affect the levels of YO-PRO-1 staining.
• Dulbecco’s Modified Eagle’s Medium (DMEM, Eurobio, cat. #CM1DME6001)
• HAM F12 medium (Eurobio, cat. #CM1H1200K)
• Minimum Essential Media (MEM, Eurobio, cat. #CM1MEM1001)
• Penicillin-streptomycin mixture (Eurobio, cat. #CABPES01-0U)
• Glutamin (Eurobio, cat. #CSTGLU00-0U)
• FCS (Eurobio, cat. #CVFSVF00-01)
• PBS without Ca^2+^ and Mg^2+^ (Eurobio, cat. #CS1PBS01K)
• Trypsin-EDTA (Eurobio, cat. #CEZTDA00-OU)
• YO-PRO-1 iodide (Thermofisher Scientific, cat. #Y3603)
**CAUTION**: YO-PRO-1 may cause skin, eye and respiratory corrosion/irritation and exerts specific target organ systemic toxicity. Avoid contact with skin and eyes and always wear gloves when preparing and using YO-PRO-1.

### Equipment

• Tissue culture incubator, humidified, 5% CO_2_/95% air, with temperature adjustable to the requirements of the cells (Binder C150)
• Microscope (DMIRB LEICA)
• Centrifuge tubes, 50 ml (Corning, cat. #430291)
• Centrifuge (Thermofisher Scientific-Jouan B4i) equipped with standard microplates holders if nonadherent cells are used
• Thoma cell counting chamber (or any cell counter)
• Black flat- and clear-bottomed 96-well tissue culture microplates (Corning, cat. #3603)
• 8-well multichannel electronic pipette 50–1200 µl (Picus, Sartorius) and tips
• Fluorescence microplate reader using dual monochromators (Safire, Tecan) with acquisition software (XFluor 4, Tecan)
• Optional: inverted fluorescence microscope

### Reagent setup

Growth culture medium for HaCaT cells Supplement DMEM medium with 10% (v/v) FCS, 2 mM glutamin and 50 IU/ml penicillin-streptomycin mixture. This medium can be stored at 4°C for up to 2 months.

Growth culture medium for HCE and ARPE-19 cells Mix DMEM and HAM F12 mediums with 1:1 ratio. Supplement the mixture medium with 10% (v/v) FCS, 2 mM glutamin and 50 IU/ml penicillin-streptomycin mixture. This medium can be stored at 4°C for up to 2 months.

Growth culture medium for JEG-3 cells Supplement MEM medium with 10% (v/v) FCS, 2 mM glutamin and 50 IU/ml penicillin-streptomycin mixture. This medium can be stored at 4°C for up to 2 months.

Maintenance medium Supplement DMEM medium, DMEM/HAM F12 mixture, or MEM with 2.5% (v/v) FCS, 2 mM glutamin and 50 IU/ml penicillin-streptomycin mixture. This medium can be stored at 4°C for up to 2 months.

Test solutions Test solutions must be prepared immediately before use, unless stability data demonstrate the acceptability of storage. The compounds suspected to activate P2X7 receptor have to be diluted in maintenance medium (for long incubation time) or in PBS without Ca^2+^ and Mg^2+^ (for short incubation time).

YO-PRO-1 staining solution Dilute YO-PRO-1 iodide stock solution to 2 µM in PBS.

**CRITICAL STEP**: The staining solution should be freshly made no more than 30 min before the cell staining step, and it should be kept in the dark at room temperature (20°C).

### Equipment setup

Using the fluorescence microplate reader software, select when possible the following measurement parameters:

• 96-well microplate (select the brand you have purchased, for example Corning)
• Excitation wavelength: 491 nm with the narrowest bandwidth (5 nm)
• Emission wavelength: 509 nm with the narrowest bandwidth (5 nm)
• Optimal gain
• Bottom reading
• Either reading with microplate cover or reading without microplate cover can be performed

## PROCEDURE

### Cell seeding • TIMING 45 min

1. Discard the growth culture medium the cells are growing in and rinse the cells by gentle agitation in PBS without Ca^2+^ and Mg^2+^ to remove any remaining serum, which might inhibit the action of the trypsin. 10 ml is enough for a 75-cm^2^ flask. Discard the PBS.
**CRITICAL STEP**: The procedures on the first day (steps 1–10) should be carried out under aseptic conditions and in the sterile environment of a laminar flow cabinet.
2. Add ~3 ml trypsin—EDTA solution at the culture temperature to the monolayer and incubate the flask in the tissue culture incubator for 2–3 min.
3. Lightly tap the flask to detach the cells and add growth culture medium (twice that of the trypsin volume to neutralize trypsin).
4. Transfer the suspension to a cell culture-treated tube and centrifuge at 1800 rpm for 3 min to eliminate the supernatant containing trypsin and resuspend the cells in fresh growth culture medium (~10 ml).
5. Count a sample of the cell suspension using a Thoma cell counting chamber (or a cell counter).
6. Dilute the cells with growth culture medium, preparing at least 15 ml per microplate of the cell suspension of an adequate cell density according to the cell type and the design of the study. Approximately 6 × 10^4^ cells/ml is adequate for most cell lines to obtain subconfluent cells in 24 h.
**CRITICAL STEP**: If nonadherent cells are used, a 5-min centrifugation step before each change of reagents is necessary.
7. Using a multichannel electronic pipette, dispense 200 µl of PBS only into the peripheral wells of a 96-well tissue culture microplate because evaporation mainly affects the peripheral wells of a plate (edge effect).
8. Agitate gently the cell suspension and place it in a sterile reservoir.
**CRITICAL STEP**: Ensure a uniform cell suspension by pipetting up and down several times.
9. Dispense 200 µl of the cell suspension into the 60 remaining wells of the microplate.
10. Cover the plate and incubate the cells in the tissue culture incubator (37°C, 5% CO_2_, humidified atmosphere) for 24 h. This incubation period allows for cell adherence and for exponential growth.

### Cell incubation with the compounds being evaluated • TIMING 30 min

11. Check the correct growth of the cells under a microscope.
12. Prepare test solutions immediately before use to avoid problems of stability and precipitation of the compounds or medium proteins.
**CRITICAL STEP**: The procedures on the second day (steps 11–15) should be carried out under aseptic conditions and in the sterile environment of a laminar flow cabinet if the incubation time with the compounds being evaluated is long (above 24 h).
13. Discard the culture medium from the microplate and rinse the cells with PBS without Ca^2+^ and Mg^2+^.
14. Add 200 µl of solutions in the wells where cells were seeded. There are several possible designs according to the study, but a very common format is: B2-G2 negative control with maintenance medium or PBS (depending on the solution being used for compounds dilution), B3-G3 positive control (ATP and/or BzATP), B4-G4 solvent control if needed, B5-G11 compounds being evaluated at different concentrations (at least three wells per concentration, so maximum 14 samples).
**CRITICAL STEP**: For long incubation times (above 24 h), the culture medium used for the dilution of the compounds being evaluated should contain less FCS than the growth culture medium to both minimize cell proliferation and the binding of the compounds to proteins, but also to assure cell survival within the exposure period.
15. Incubate the microplate at appropriate conditions (room temperature or 37°C, short or long incubation times).

### YO-PRO-1 uptake assay • TIMING 15 min

16. After incubation with the compounds being evaluated for P2X7 receptor activation, discard the supernatant and rinse the cells with PBS without Ca^2+^ and Mg^2+^.
**CRITICAL STEP**: Before performing the YO-PRO-1 uptake assay, check that cell viability is not altered by the products being studied with the neutral red assay for example, as described by Repetto *et al*. [49] If the products being studied statistically decreased cell viability, then lower concentrations should be tested to identify subcytotoxic concentrations for the YO-PRO-1 experiment.
17. Add 200 μl of YO-PRO-1 staining solution to all cell-seeded wells and incubate at room temperature for 10 min in the dark.
**CRITICAL STEP**: It is important to keep the microplate in the dark during incubation with YO-PRO-1. Aluminum foil can be used to entirely recover the microplate.

### Data acquisition by microplate cytometry • TIMING 1 min

18. Measure the fluorescence signal in a fluorescence microplate reader with YO-PRO-1 measurement parameters (see Equipment setup).
19. Analyze data using the software provided with the microplate reader to calculate mean fluorescence intensity, standard deviation and draw graphs.
20. It is recommended to repeat the experiment at least three different times.

### Optional: Observation by live-cell fluorescence microscopy • TIMING 30 min

21. Discard the YO-PRO-1 solution
22. Observe YO-PRO-1 fluorescence using live-cell fluorescence microcopy and take pictures to illustrate YO-PRO-1 uptake.

## ANTICIPATED RESULTS

The procedure describes a model system using the human epithelial HCE, ARPE-19, HaCaT, JEG-3 cells, and P2X7 receptor agonists, ATP and BzATP, to exemplify this approach for evaluating P2X7 receptor activation. In the four cell lines, neither ATP nor BzATP caused decrease in cell viability. To analyze YO-PRO-1 fluorescence data, we suggest to calculate mean fluorescence intensity of negative control (*n* = 6 for one experiment), then to normalize each fluorescence raw data (negative control, positive control, solvent, and compounds being evaluated) to the fluorescence mean of negative control (divide all individual value for each sample by the mean value of negative control). Then, means and standard deviations of each sample are calculated from normalized data. In our case, statistical analysis was performed using GraphPad Prism 6 software to run a one-way ANOVA follower by Dunnett’s test with risk α at 5%.

**Figure 4.**
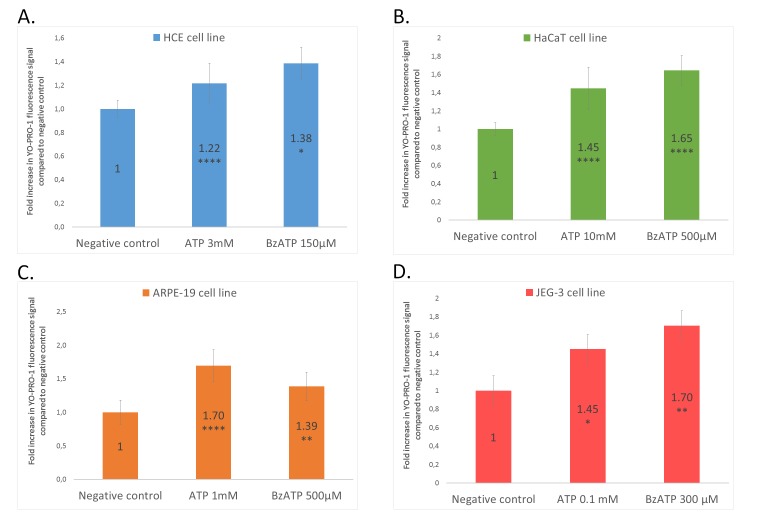
**YO-PRO-1 uptake assay on human corneal cells (A, HCE), human cutaneous cells (B, HaCaT), human retinal cells (C, ARPE-19) and human placental cells (D, JEG-3).** ATP and BzATP were incubated for 15 min then the assay was carried out. Graphs show the fluorescence mean with the standard deviations. *P < 0.05; **P < 0.01; ****P < 0.0001 compared to negative control.

**Figure 4** shows the results obtained with human corneal, cutaneous, retinal and placental cell lines. The cells were incubated with ATP or BzATP for 15 min and the assay was then carried out. An increase in YO-PRO-1 uptake is observed with both ATP and BzATP.

In **Figure 4A**, when HCE cells are incubated with ATP 3 mM and BzATP 150 µM, we respectively observe a 1.22-fold and a 1.38-fold increase in YO-PRO-1 uptake, compared to negative control (arbitrary fixed to 1). To illustrate these data, the cells were observed under fluorescence microscopy and representative pictures were taken (see **Fig. S1**). In **Figure 4B**, ATP 10 mM and BzATP 500 µM respectively induce a 1.45-fold and a 1.65-fold increase in YO-PRO-1 uptake, compared to negative control, in HaCaT cells. In **Figure 4C**, a 1.45-fold increase in YO-PRO-1 uptake after ATP 10 mM and a 1.65-fold increase after BzATP 500 µM are observed in ARPE-19 cells. In **Figure 4D**, ATP 0.1 mM induced a 1.45-fold increase in YO-PRO-1 in JEG-3 cells compared to negative control, and BzATP 300µM induced a 1.70-fold increase. It is important to know that some compounds may induce higher YO-PRO-1 intake than ATP and BzATP do [37, 46].

The assay has to be performed on cells expressing P2X7 receptor; otherwise, negative results could be misinterpreted and the conclusions of YO-PRO-1 uptake assay would be false.

In conclusion, the procedure we propose here using microplate cytofluorometry is easier and requires less handling steps than other fluorescence techniques. 96-well plate based YO-PRO-1 assay is a reproducible and fast method to study both P2X7 receptor activation by toxic agents at subnecrotic concentrations and P2X7 receptor inhibition by antagonists.

## TROUBLESHOOTING

Troubleshooting advice can be found in **Table 1**.

**Table 1.** Troubleshooting table.

Problem	Possible reason	Solution
Low YO-PRO-1 signal in positive control	Low cell density	Increase cell density
	YO-PRO-1 exposure to light	Use aluminum foils to protect the microplate and the recipient where YO-PRO-1 staining solution is prepared
	Alteration of YO-PRO-1 dye	Avoid freeze/thaw cycles by aliquoting stock solution
	Cell of interest may not express P2X7 receptor	Check P2X7 expression using immunolabelling
	Staining solution was prepared too much in advance	Prepare YO-PRO-1 staining solution immediately before cell staining
High YO-PRO-1 signal in negative control	Wide bandwidth	Reduce bandwidth

## Abbreviations used

ATP: adenosine triphosphate
FCS: fetal calf serum

## Supplementary information

**Figure S1**. Fluorescence microscopy observations of YO-PRO-1 uptake assay on HCE cells: Negative control (A), ATP 3mM (B) and BzATP 150µM (C) related to **Figure 4A**. ATP and BzATP were incubated for 15 min then the assay was carried out. The cells were observed under fluorescence microscopy (Leica DMIRB, Wetzlar, Germany) and representative pictures were taken using Nikon Coolpix camera (Nikon, Shinjuku, Japan).
